# CXC chemokine ligand‐13 promotes metastasis via CXCR5‐dependent signaling pathway in non‐small cell lung cancer

**DOI:** 10.1111/jcmm.16743

**Published:** 2021-08-24

**Authors:** Chia‐Chia Chao, Wei‐Fang Lee, Shih‐Wei Wang, Po‐Chun Chen, Ayaho Yamamoto, Tsung‐Ming Chang, Shun‐Long Weng, Ju‐Fang Liu

**Affiliations:** ^1^ Department of Respiratory Therapy Fu Jen Catholic University New Taipei City Taiwan; ^2^ School of Dental Technology College of Oral Medicine Taipei Medical University Taipei Taiwan; ^3^ Institute of Biomedical Sciences MacKay Medical College New Taipei City Taiwan; ^4^ Department of Medicine MacKay Medical College New Taipei City Taiwan; ^5^ Graduate Institute of Natural Products College of Pharmacy Kaohsiung Medical University Kaohsiung Taiwan; ^6^ Translational Medicine Center Shin‐Kong Wu Ho‐Su Memorial Hospital Taipei City Taiwan; ^7^ Department of Biotechnology College of Medical and Health Science Asia University Taichung Taiwan; ^8^ Department of Medical Research China Medical University Hospital China Medical University Taichung Taiwan; ^9^ Child Health Research Centre The University of Queensland South Brisbane Qld Australia; ^10^ Institute of Physiology School of Medicine National Yang Ming Chiao Tung University Taipei City Taiwan; ^11^ Department of Obstetrics and Gynecology Hsinchu MacKay Memorial Hospital Hsinchu City Taiwan; ^12^ School of Oral Hygiene College of Oral Medicine Taipei Medical University Taipei City Taiwan

**Keywords:** CXCL13, CXCR5, lung cancer, metastasis, VCAM‐1

## Abstract

The CXC chemokine ligand‐13 (CXCL13) is a chemoattractant of B cells and has been implicated in the progression of many cancers. So far, CXCL13 and its related receptor CXCR5 have been proved to regulate cancer cell migration as well as tumour metastasis. However, the role of CXCL13‐CXCR5 axis in metastasis of lung cancer is still poorly understood. In this study, we found that CXCL13 and CXCR5 were commonly up‐regulated in lung cancer specimens compared with normal tissues among different cohorts. Our evidence showed that CXCL13 obviously promoted migration of lung cancer cells, and this effect was mediated by vascular cell adhesion molecule‐1 (VCAM‐1) expression. We also confirmed that CXCR5, the major receptor responsible for CXCL13 function, was required for CXCL13‐promoted cell migration. We also test the candidate components which are activated after CXCL13 treatment and found that phospholipase C‐β (PLCβ), protein kinase C‐α (PKCα) and c‐Src signalling pathways were involved in CXCL13‐promoted cell migration and VCAM‐1 expression in lung cancer cells. Finally, CXCL13 stimulated NF‐κB transcription factor in lung cancer cells, contributing to VCAM‐1 expression in translational level. These evidences propose a novel insight into lung cancer metastasis which is regulated by CXCL13.

## 1 INTRODUCTION

Lung cancer is the major cause responsible for cancer death worldwide. Most of lung cancer patients are diagnosed as non‐small cell lung cancer (NSCLC), including lung adenocarcinoma (LUAD) and lung squamous‐cell carcinoma (LUSC).^1^ The golden treatment regimens such as chemotherapy and radiotherapy have improved prognosis of lung cancer over the past decades, survival rate remains low; the 5‐year survival rate is about 15% while the patients are diagnosed with lung cancer. The low survival rate of lung cancer is caused by distant metastasis occurred in late‐stage. However, the mechanisms of metastasis in NSCLC have not yet been fully illustrated. Cancer metastasis is a complex, multi‐step process, in which cells escape from the primary origin and form metastases at a distant site.^2^ Primary cancer cells loss their cell adhesion phenotype, polarity, rearrange cytoskeleton and adjacent extracellular matrix (ECM) to invade the surrounding stroma.^3^

Primary tumour release cancer cells into the circulation long before diagnosis. To establish disseminated cancer cells that may finally progress to metastases, circulating cancer cells must first transmigrate endothelial capillary walls and successfully adapt to the new environmental stress.^4^ Transendothelial migration (TEM) of monocytes is the process by which monocytes or leukocytes leave the circulatory system and extravasate through the endothelial lining of the blood vessel wall, then enter the underlying tissue.^5^ Similar to leukocyte extravasation, lung metastasis requires cancer cells to cross the lung endothelium.^6^ Vascular cell adhesion molecule 1 (VCAM‐1) has been implicated in early leukocyte transmigration.^5^ More and more is being learnt about the key role played by VCAM‐1 in tumorigenesis and metastasis.^7^ VCAM‐1 expression correlates with glioblastoma grade,^8^ as well as with poor survival in lung cancer ^9^ and epithelial ovarian cancer.^10^ Previous research has indicated that VCAM‐1 contributes to breast cancer cells metastasizing to lung.^11^ Moreover, VCAM‐1 overexpression in breast cancer promotes bone metastasis by activating osteoclastogenesis and subsequent early relapse.^12^ These findings establish VCAM‐1 as a promising target for the prevention and inhibition of metastasis.

Several studies have reported that chemokines have a key role in regulating the homing of leukocytes as well as cancer cells.^13^ We found that expression levels of CXCL13 and CXCR5 were highly correlated with lung cancer progression.^14^ Chemokines have revealed that they have crucial roles in the metastasis and progression of different cancers. In breast cancer, the CXCL12/CXCR4 axis has been extensively investigated in regard to the homing of cancer cells to metastatic sites and promoting metastasis.^15^, ^16^ Metastatic breast cancer cells express high levels of CXCR4; its ligand CXCL12 is highly secreted by stromal cells within these tissues.^17^ CXCL13, also known as B‐cell‐attracting chemokine 1 (BCA‐1), and its receptor CXCR5 help to regulate lymphocyte migration and promote inflammation.^18^, ^19^ The binding of CXCL13 to CXCR5 elicits the activation of PI3K/Akt, MAPK, integrin‐β3/Src/FAK and the DOCK2/Rac/JNK pathways, which regulate cell survival, invasion, and proliferation, respectively.^20^

Recently, the CXCL13‐CXCR5 axis has been implicated in the progression of various tumours, including colon cancer,^21^ hepatocellular carcinoma,^22^ chronic lymphocytic leukaemia,^23^ breast cancer,^23^ neuroblastoma ^24^ and prostate cancer.^25^ Much clinical evidence highlights the crucial roles of the CXCL13‐CXCR5 axis in the regulation of tumour growth, progression and metastasis in the tumour microenvironment.^26^ However, the role of the CXCL13‐CXCR5 axis in lung cancer is poorly discussed.

Here, we found that expression levels of CXCL13 and CXCR5 were highly correlated with lung cancer progression. Also, CXCL13/CXCR5 axis contributed to cell motility in lung cancer cells, which was caused by VCAM‐1 expression. Finally, PLCβ, PKCα and c‐Src signalling pathways were involved in CXCL13‐promoted cell migration and VCAM‐1 expression in lung cancer cells. Our present work provides novel application of CXCL13/CXCR5 axis in lung cancer therapeutic strategy.

## 2 MATERIALS AND METHODS

### 2.1 Meta‐analysis of associations between CXCL13, CXCR5 and VCAM‐1 expression in NSCLC

The data sets used in meta‐analysis were selected from Lung Cancer Explorer (LCE; http://qbrc.swmed.edu/lce/), which contains data sets of gene profiling of tumour patients and healthy subjects obtained from Gene Expression Omnibus (GEO), The Cancer Genomics Atlas (TCGA) and other published literatures. Data sets of lung cancer tissues containing adenocarcinoma (LUAD) and squamous‐cell carcinoma (LUSC) were selected from LCE. For the meta‐analysis of CXCL13 and CXCR5 expression levels in selected data sets, the sample size of each group (ADC, SCC, normal) more than 20 was required to assure robust analysis results. The correlation analysis of CXCL13, CXCR5 and VCAM‐1 were performed with Gene Expression Profiling Interactive Analysis (GEPIA; http://gepia.cancer‐pku.cn/) web‐based tool.^27^ The RNA profiling data of CXCL13, CXCR5 and VCAM‐1 were performed with Correlation Analysis provided by GEPIA.

### 2.2 Materials

Anti‐mouse and anti‐rabbit IgG‐conjugated horseradish peroxidase, rabbit polyclonal antibodies specific for PLCβ3 (Santa Cruz, sc‐403), PKCα(Santa Cruz, sc‐208), c‐Src (cell signaling, #2109s), p65 (Santa Cruz, sc‐109), IκBα (Santa Cruz, sc‐1643), p‐PLCβ (cell signaling, #2481s), p‐PKCα(cell signaling, #9375), p‐c‐Src (cell signaling, #5473), p‐p65 (cell signaling, #3033), p‐IKKα/β (cell signaling, #2694), IKKα/β (Santa Cruz, sc‐7218), p‐IκBα (cell signaling, #9246), CXCL13 (GeneTex, GTX108471), CXCR5 (GeneTex, GTX100351), VCAM‐1 (GeneTex, GTX110684) and β‐Actin (GeneTex, GTX109639) were purchased from Cell Signaling Technology, Inc, Santa Cruz Biotechnology, Inc and GeneTex Inc. All other chemicals were obtained from Sigma‐Aldrich. The CXCL13 shRNA plasmid was purchase from RNAiCore (Clone ID: TRCN0000057983). All siRNAs were purchased from Sigma‐Aldrich (Mission^®^ siRNA).

### 2.3 Cell culture

CL1‐0 lung cancer cell line was provided by Dr Shun‐Fa Yang (Institute of Medicine, Chung Shan Medical University). The cells will be cultured in α‐MEM medium supplemented with 20 mmol/L HEPES, 10% FBS, 2 mmol/L glutamine, penicillin and streptomycin (Invitrogen) and maintained at 37°C in a 5% CO_2_ atmosphere.

### 2.4 Cell viability assays

Cells (7 × 10^3^) were seeded in 96‐well plates prior to the indicated treatment. Cell viability was assayed using CCK8 reagent as described ^28^ and was determined as the percentage of the control. Samples were protected from light during the assays, and the incubation time for CCK8 was set to 4 hours. Each condition was performed in eight replicate wells, and the data present were from at least three independent experiments.

### 2.5 Transwell migration and invasion assay

All cell migration and invasion assays were performed using Transwell inserts (8‐μm pore size; Costar) in 24‐well plates. Lung cancer cells were pre‐treated for 30 minutes with the indicated concentrations of inhibitors or vehicle (0.1% DMSO). Cells (3 × 10^4^ in 200 μL of serum‐free medium) were then seeded in the upper chamber of the Transwell chamber, and 300 μL of the same medium containing varying concentrations of CXCL13 (R&D Systems) was placed in the lower chamber. After 24 hours, the migrated cells were fixed in 3.7% formaldehyde for 15 minutes and stained with 0.05% crystal violet in PBS for 15 minutes. The cells beneath the filters were removed and rinsed by PBS. The migrated cells on the underside of the filters were examined and counted by using light microscopy. For the invasion assay, Transwell inserts were coated with BD Matrigel (BD Biosciences) before use. The experimental protocol for the invasion assay was as described above for the cell migration assay. Each experiment was performed with triplicate wells and was repeated at least three times.

### 2.6 Western blot analysis

The cellular lysates collected from lung cancer cells were resolved on SDS–PAGE and transferred to Immobilon polyvinyldifluoride (PVDF) membranes. The blots were blocked with NAP‐BLOCKER^™^ in TBS [2X] (G‐Biosciences) for 1 hour at room temperature and then probed with rabbit anti‐human antibodies against (1:1000) for 1 hour at room temperature. After three washes, the blots were subsequently incubated with a donkey anti‐rabbit peroxidase‐conjugated secondary antibody (1:5000) for 1 hour at room temperature. The blots were visualized with enhanced chemiluminescence substrate and photographed using a charge‐coupled device camera‐based detection system (UVP Inc). Quantitative data were obtained using ImageJ software (National Institutes of Health).

### 2.7 Quantitative real‐time PCR

qPCR analysis was carried out using TaqMan^®^ one‐step PCR Master Mix (Applied Biosystems). One hundred ng of total cDNA was added per 25‐µL reaction with sequence‐specific primers and TaqMan^®^ probes. Sequences for all target gene primers and probes were purchased commercially (β‐actin was used as internal control; Applied Biosystems). Quantitative RT‐PCR assays were carried out in triplicate on StepOnePlus sequence detection system. The cycling conditions were 10‐minutes polymerase activation at 95°C followed by 40 cycles at 95°C for 15 s and 60°C for 60 s. The threshold was set above the non‐template control background and within the linear phase of target gene amplification to calculate the cycle number at which the transcript was detected (denoted as C_T_).

### 2.8 Immunofluorescence

Cells were seeded on chamber slides before immunofluorescence staining. After treatment with indicated condition as described in figure legends, the cells were washed with PBS and fixed in 3.7% formaldehyde for 10 minutes at room temperature. Cells were washed three times with PBS and blocked with 4% BSA for 15 minutes. Cells were then incubated with the p65 primary antibody (1:100) for 1 hour at room temperature, washed again and incubated with FITC‐conjugated secondary antibody (1:100) for 1 hour. Finally, cells were washed, mounted with DAPI containing solution, and photographed with a Nikon Ti2 microscopy System (Nikon).

### 2.9 Transfection of shRNAs and siRNAs

The lung cancer cells were transfected with shRNAs or siRNAs using Lipofectamine 3000 (Invitrogen) according to the manufacturer's recommendations. After 24 hours post‐transfection, the cells were performed with cell migration, qPCR, Western blot, Immunofluorescence or luciferase reporter assays as described in Figure Legends section.

### 2.10 Luciferase reporter assay

The luciferase activity in the transfected cells was measured using a Luciferase Reporter Assay System (Promega) according to the manufacturer's instructions. Lung cancer cells were transfected with NF‐κB vectors using Lipofectamine 3000 (Invitrogen) for 24 hours, the cells were treated with inhibitors or vehicle control for 30 minutes and then exposed to CXCL13 (30 ng/mL) for 24 hours. Transactivation was determined by monitoring the firefly luciferase levels in the pGL2 vector. The luciferase assay was performed by adding lysis buffer (100 μL) and harvesting the cells through centrifugation (16200 *g* for 5 minutes). The supernatant was transferred to fresh tubes, and 20 μL of cell lysate was added to 80 μL of fresh luciferase assay buffer in an assay tube. The luciferase activity was measured using a microplate luminometer. Luciferase activity was normalized to transfection efficiency based on the cotransfected β‐galactosidase expression vector.

### 2.11 Statistics

The values given will be the means ± SD The significance of difference between two experimental groups was analysed by Student's *t* test, and multiple group comparisons were performed using one‐way analysis of variance (one‐way ANOVA) with Fisher LSD post hoc tests. Between‐group differences will be considered significant if the *P* value is <.05.

## 3 RESULTS

### 3.1 CXC chemokine ligand‐13 and CXCR5 is highly expressed in lung cancer specimens

**FIGURE 1:**
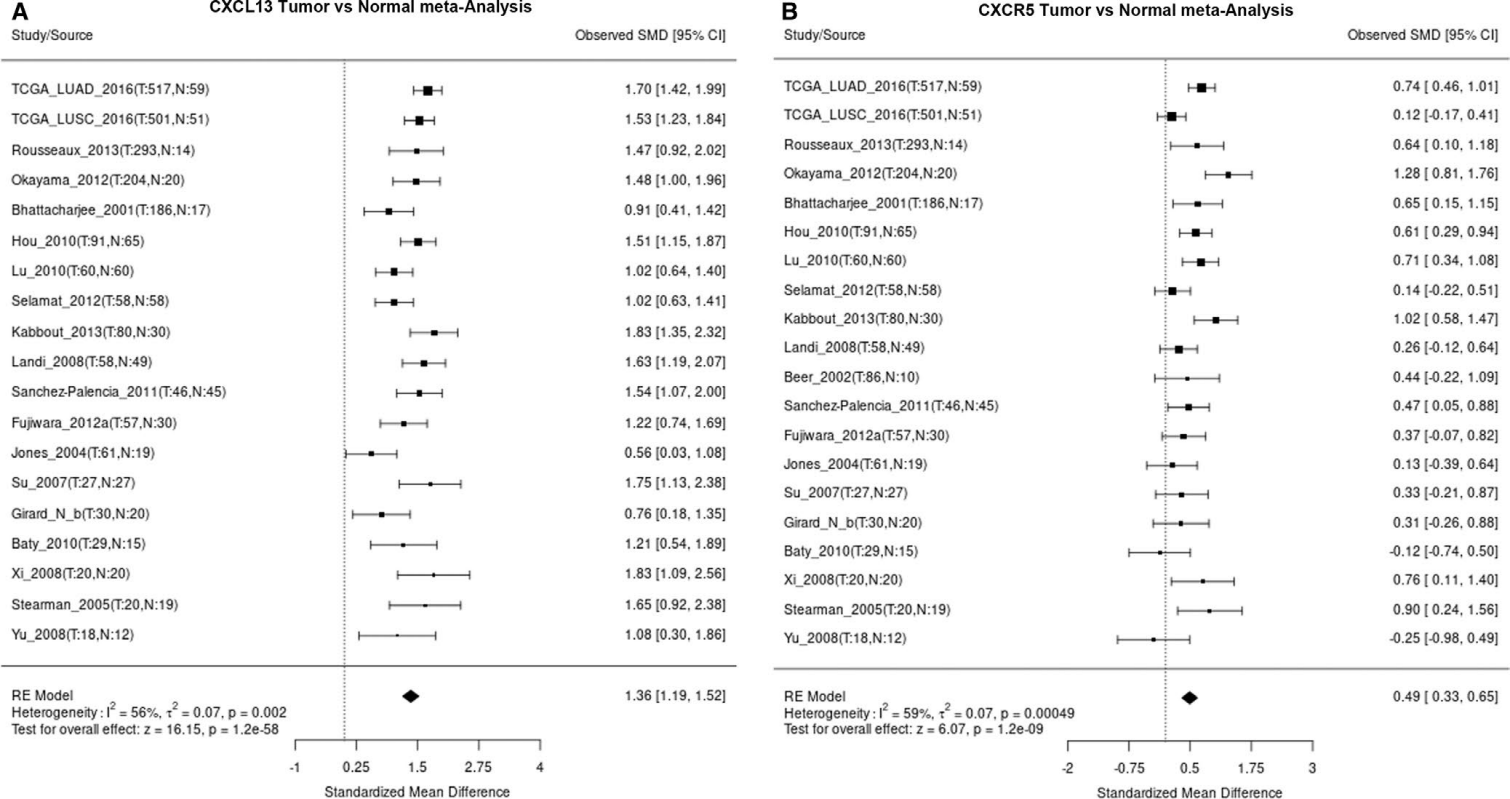
Tumour versus normal expression difference of CXCL13 and CXCR5 in LUAD and LUSC. A and B, Expression patterns of CXCL13 and CXCR5 in all lung cancer tissues containing LUAD and LUSC were analysed by using LCE tool and showed by forest plot. SMD: standardized mean difference

CXC chemokine ligand‐13 has been implicated in the progression of various cancers.^26^ The evidence also shows that the CXCL13‐CXCR5 pathway is important in prostate cancer cell invasion and migration.^25^ However, little is known about the CXCL13‐CXCR5 axis and its roles in lung cancer. We first examined the expression profile of CXCL13 and CXCR5 in lung cancer tissues by using online lung cancer‐specific database‐the Lung Cancer Explorer (LCE).^29^ Surprisingly, with meta‐analysis in all lung cancer tissues containing adenocarcinoma (LUAD) and squamous‐cell carcinoma (LUSC), we found consistent up‐regulation of CXCL13 and CXCR5 in multiple lung cancer studies (Figure 1A,B), suggesting the impact of CXCL13‐CXCR5 axis in lung cancer progression.

### 3.2 CXC chemokine ligand‐13 promotes cell mobility in lung cancer cells

**FIGURE 2:**
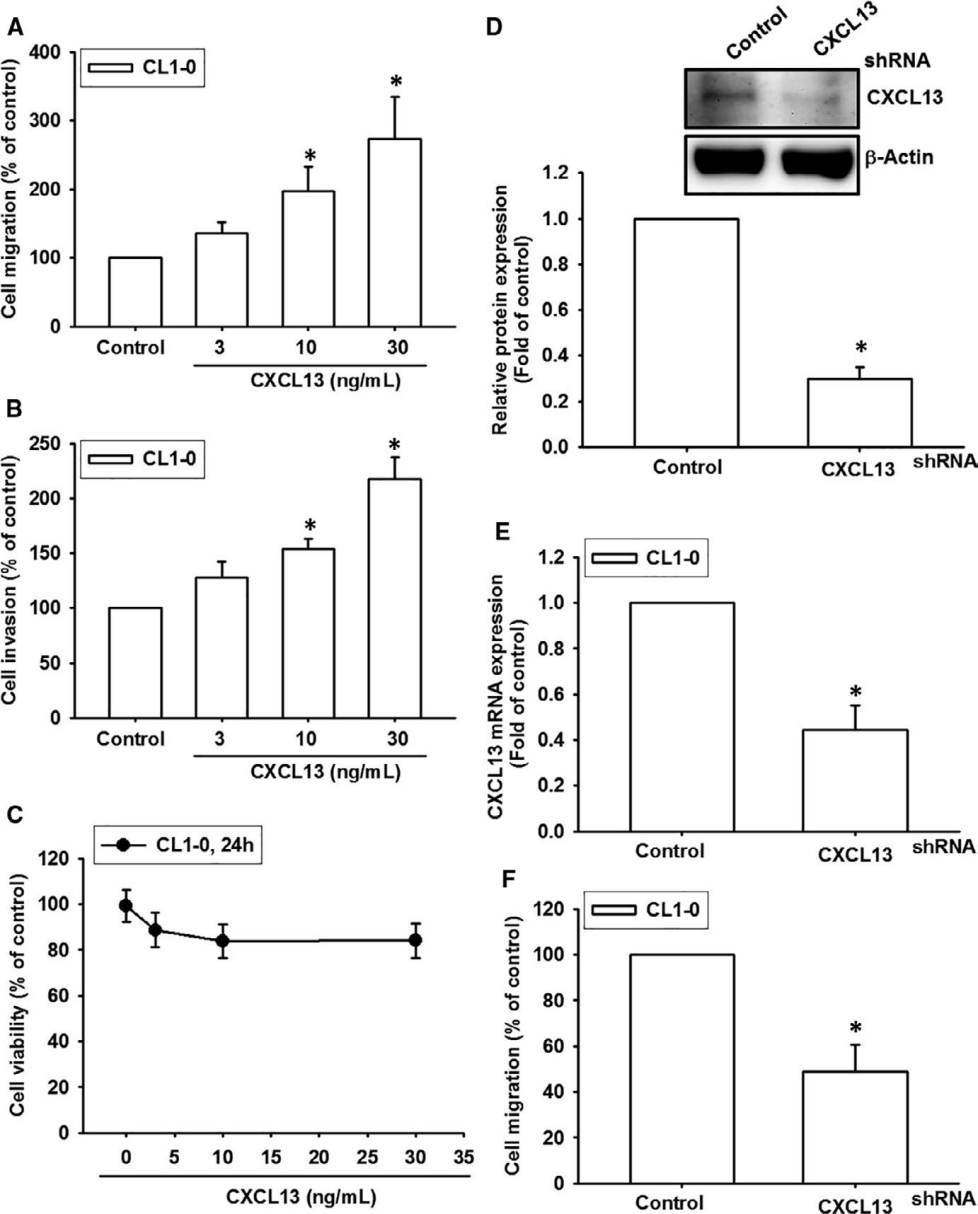
CXC chemokine ligand‐13 promotes cell mobility in lung cancer cells. (A‐C) The CL1‐0 lung cancer cells were incubated with various concentrations (0, 3, 10 and 30 ng/mL) of human recombinant CXCL13 for 24 h, and the migration A, or invasion assay B, were performed by using Transwell. The migrating and invading cells were counted by microscope. C, The cell viability of CL1‐0 cells in response to CXCL13 incubation was assessed by CCK8 reagent. D and E, The CL1‐0 cells were transfected with CXCL13 shRNA for 24 h, and the knockdown efficiency of CXCL13 was confirmed by Western blot and qPCR. F, The CL1‐0 transfected with CXCL13 was subjected to cell migration assay. Results are expressed as the mean ± SD of triplicate samples. *P < .05 compared to the control group

To determine whether CXCL13 affected migration of lung cancer cells, a Transwell migration and invasion assays were used. The results indicated treatment with recombinant CXCL13 dramatically increased migration and invasion of CL1‐0 lung cancer cells (Figure 2A,B). The cell viability assay showed no statistical significance after CXCL13 incubation in lung cancer cells (Figure 2C). With the gene knockdown by CXCL13 shRNA in lung cancer cells, we confirmed that expression of CXCL13 was contributed to cell migratory ability in lung cancer cells (Figure 2D‐F).

### 3.3 Vascular cell adhesion molecule‐1 expression contributes to CXCL13‐induced cell migration in lung cancer cells

**FIGURE 3:**
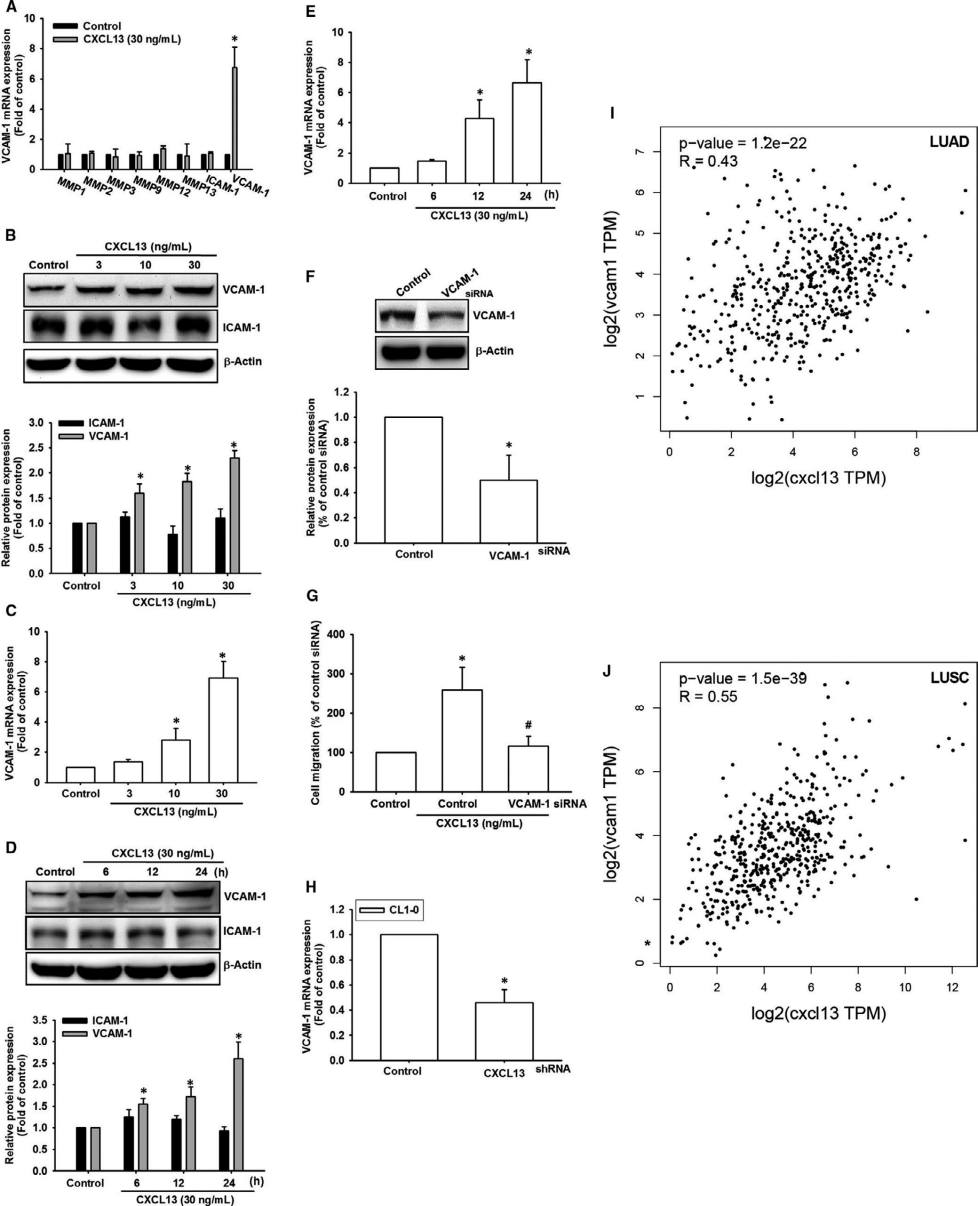
Vascular cell adhesion molecule‐1 expression is responsible for CXCL13‐promoted cell migration in lung cancer. A, The screening of candidate molecules associated with cell migration ability. The CL1‐0 lung cancer cells were incubated with CXCL13 (30 ng/mL) for 24 h, follow by collecting RNA sample, then subjecting to qPCR analysis to validate gene expression of candidate molecules which associated with cell migration ability. B and C, The CL1‐0 lung cancer cells were incubated with different concentrations (0, 3, 10 and 30 ng/mL) of CXCL13 for 24 h, the RNA or cell lysate were collected, then subjected to analyse gene expression level of VCAM‐1 by qPCR and Western blot. D and E, The CL1‐0 lung cancer cells were treated with CXCL13 (30 ng/mL) for different time interval (0, 6, 12 and 24 h), the RNA or cell lysate were collected and then subjected to analyse gene expression level of VCAM‐1 by qPCR and Western blot. F, The CL1‐0 lung cancer cells were transfected with VCAM‐1 siRNA for 24 h, and then, the expression level of VCAM‐1 was confirmed by Western blot. G, The CL1‐0 lung cancer cells transfected with VCAM‐1 siRNA were subjected to analyse cell mobility in the presence of CXCL13 (30 ng/mL) by Transwell migration assay. H, The CL1‐0 lung cancer cells transfected with CXCL13 shRNA were performed with qPCR to detect VCAM‐1 expression level. I and J, The correlation analysis data of CXCL13 and VCAM‐1 by using GEPIA bioinformatics tool. The boxplot analysis used log_2_ (TPM + 1) for log‐scale. Results are expressed as the mean ± SD of triplicate samples. *P < .05 compared to the control group. #*P* < .05 compared to the CXCL13‐treated group

We next explored the candidate molecules that regulated cell migration in lung cancer cells. The data showed that VCAM‐1 was obviously induced after incubation with CXCL13 (Figure 3A). Furthermore, CXCL13‐promoted VCAM‐1 expression in lung cancer cells in a dose‐ and time‐dependent manners (Figure 3B‐E). Knockdown of VCAM‐1 expression in lung cancer cells confirmed that CXCL13‐promoted cell migration was mediated through VCAM‐1 (Figure 3F‐H). Finally, we manipulated online gene expression profiling tool GEPIA ^27^ to assess the correlation between CXCL13 and VCAM‐1 in lung cancer tissues. The data indicated that VCAM‐1 expression levels were highly correlated with CXCL13 in specimens of LUAD and LUSC (Figure 3I,J). In summary, these evidences propose that VCAM‐1 is required for cell migratory potential in response to CXCL13 incubation.

### 3.4 CXCR5 mediates cell migration and VCAM‐1 expression in response to CXCL13 incubation in lung cancer cells

**FIGURE 4:**
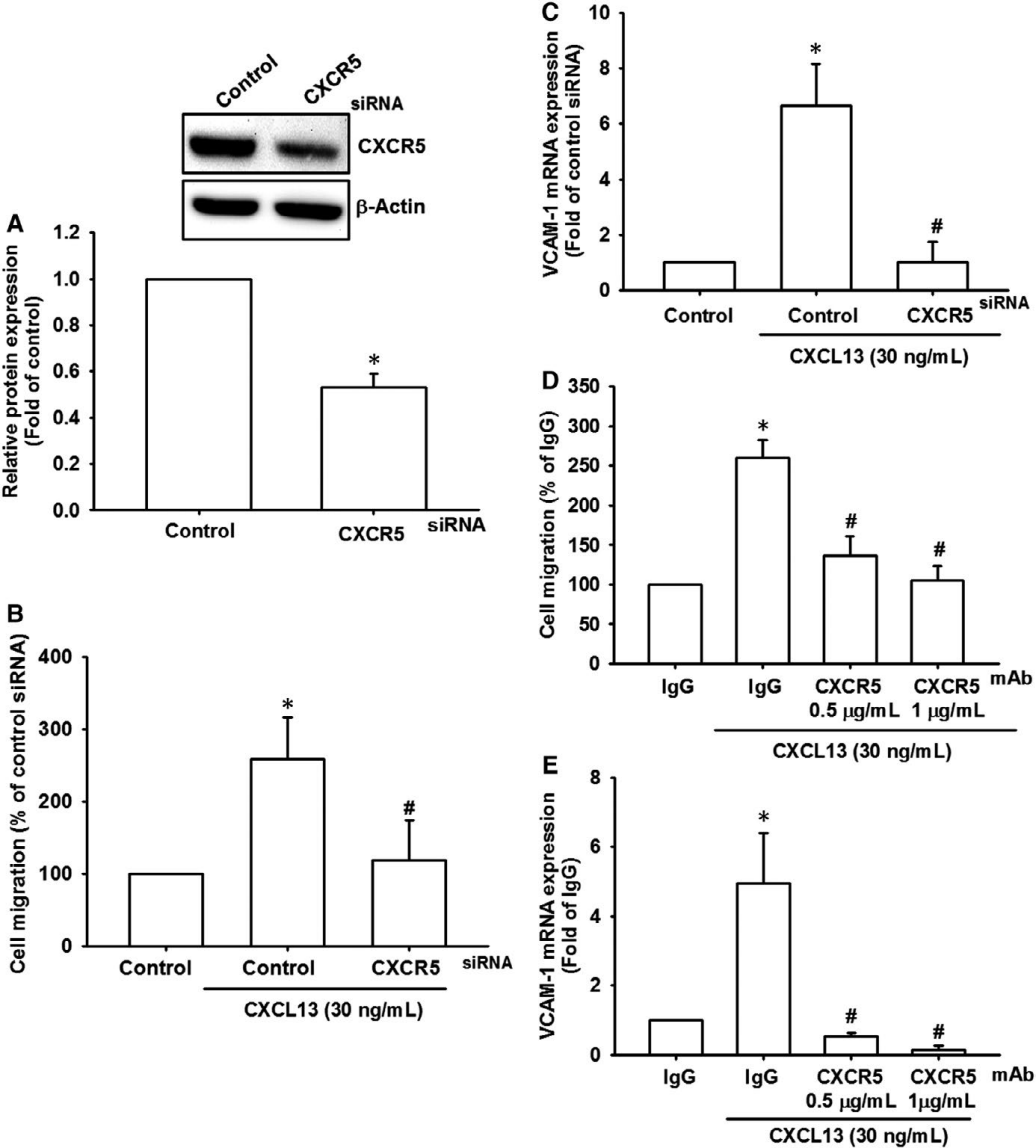
CXCR5 mediates the VCAM‐1 expression and cell migration in response to CXCL13 incubation. A, The CL1‐0 lung cancer cells were transfected with CXCR5 siRNA for 24 h, and the knockdown efficiency of CXCR5 was confirmed by Western blot. B and C, The CL1‐0 lung cancer cells transfected with CXCR5 siRNA were further performed with cell migration assay B, in the presence of CXCL13 (30 ng/mL) for 24 h, as well as qPCR C, to detect VCAM‐1 expression. D and E, The CL1‐0 lung cancer cells were pre‐incubated with CXCR5 neutralized antibody (0.5 and 1 μg/mL) for 1 h and then performed with cell migration assay D, and qPCR E, in the presence of CXCL13 (30 ng/mL) for 24 h to evaluate cell migratory ability and VCAM‐1 expression, respectively. Results are expressed as the mean ± SD of triplicate samples. *P < .05 compared to the control group. #*P* < .05 compared to the CXCL13‐treated group

CXCR5, a member of the G‐protein coupled receptors (GPCR) family, is required for CXCL13 biological functions.^30^ The CXCL13‐CXCR5 axis is implicated in regulating lymphocyte migration and promoting inflammation.^18^, ^19^ Therefore, we hypothesized that CXCL13‐induced lung cancer cell migration may be regulated by CXCR5. Lung cancer cells transfected with a CXCR5 siRNA markedly abolished CXCL13‐induced cell migration and VCAM‐1 expression (Figure 4A‐C). Meanwhile, CXCR5 neutralized antibody also dose‐dependently reversed cell migration and VCAM‐1 expression in CXCL13‐treated lung cancer cells (Figure 4D,E). Conclusively, these results identify the crucial role of CXCR5 receptor in regulating cell migration and VCAM‐1 expression, which caused by CXCL13 incubation in lung cancer cells.

### 3.5 Phospholipase C‐β/PKCα/c‐Src signalling pathways are required for CXCL13‐promoted cell migration and VCAM‐1 in lung cancer cells

**FIGURE 5:**
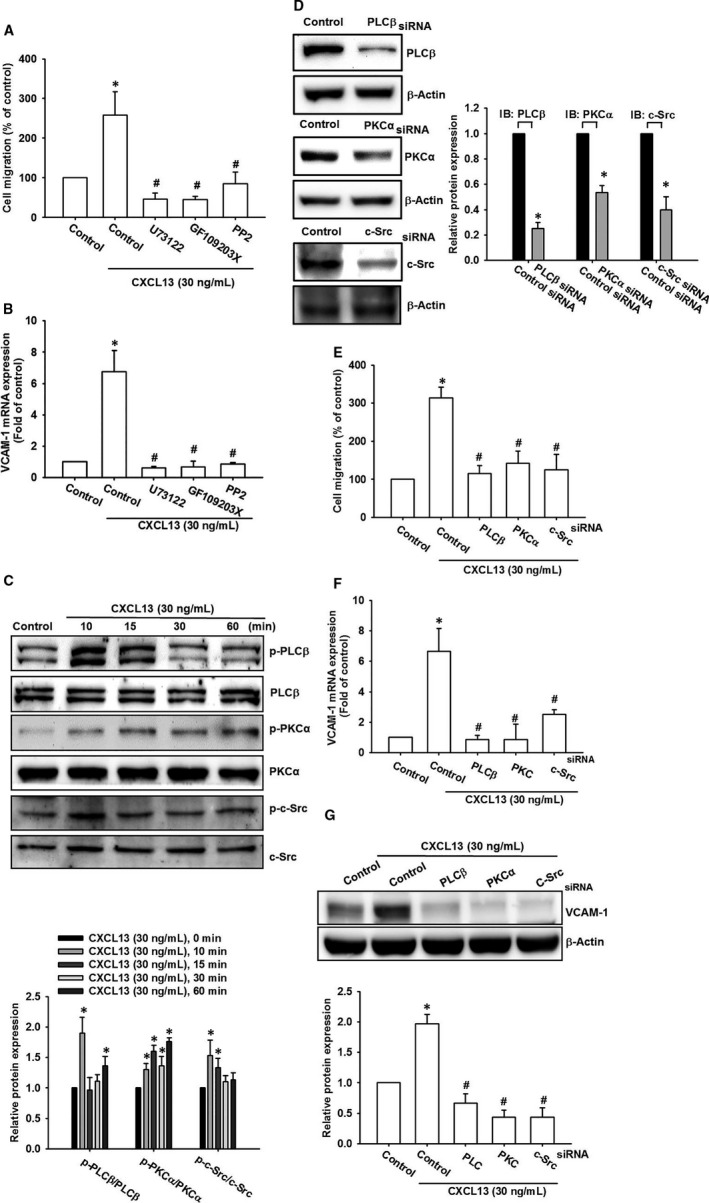
Phospholipase C‐β/PKCα/c‐Src signalling pathways are required for CXCL13‐promoted cell migration and VCAM‐1 in lung cancer cells. A and B, The CL1‐0 lung cancer cells were pre‐treatment with pathway inhibitors of PLCβ, PKCα or c‐Src (U73122, 0.5 μmol/L; GF109203X, 3 μmol/L; PP2, 5 μmol/L) for 1 h and then performed with cell migration assay A, and qPCR B, in the presence of CXCL13 (30 ng/mL) for 24 h to evaluate cell migratory ability and VCAM‐1 expression, respectively. C, The CL1‐0 lung cancer cells were incubated with CXCL13 (30 ng/mL) for different time course (0, 10, 15, 30 and 60 min), the cell lysates were collected, and activation of PLCβ, PKCα and c‐Src signalling pathways was evaluated by monitoring phosphorylation of these proteins. The total proteins were used as internal control. D, The CL1‐0 lung cancer cells were transfected with PLCβ, PKCα or c‐Src siRNAs for 24 h, and the knockdown efficiency was confirmed by using Western blot. E, The CL1‐0 lung cancer cells transfected with PLCβ, PKCα or c‐Src siRNAs were further evaluated cell migration ability E, and VCAM‐1 expression F and G, by using Transwell migration assay, qPCR and Western blot, respectively, in the presence of CXCL13 (30 ng/mL) for 24 h. Results are expressed as the mean ± SD of triplicate samples. *P < .05 compared to the control group. #*P* < .05 compared to the CXCL13‐treated group

The intracellular signalling pathways that activate transcription factors, and subsequently up‐regulate gene expression, are crucial for cellular biological functions. The chemokine receptor elicits multiple signalling pathways including the PLC/PKC, c‐Src, JAK/STAT, PI_3_K/Akt and MAPK pathways.^31^ Here, we investigated whether CXCL13 activated these signalling pathways in lung cancer cells. The results indicated that pre‐treatment with PLCβ, PKCα and c‐Src inhibitors (U73122, GF109203X and PP2) reversed CXCL13‐promoted cell migration and VCAM‐1 in lung cancer cells (Figure 5A,B). Moreover, incubation with CXCL13 obviously induced phosphorylation of PLCβ, PKCα and c‐Src (Figure 5C). Finally, we certified our finding by using siRNA to suppress these pathways activation, and the data showed that transfection with PLCβ, PKCα and c‐Src siRNA apparently reversed cell migration and VCAM‐1 expression after CXCL13 incubation (Figure 5D‐G). These results suggest that the PLCβ/PKCα/c‐Src signalling pathways contribute to CXCL13‐promoted cell migration in lung cancer cells.

### 3.6 NF‐κB transcription factor is responsible for CXCL13‐induced cell migration in lung cancer cells

**FIGURE 6:**
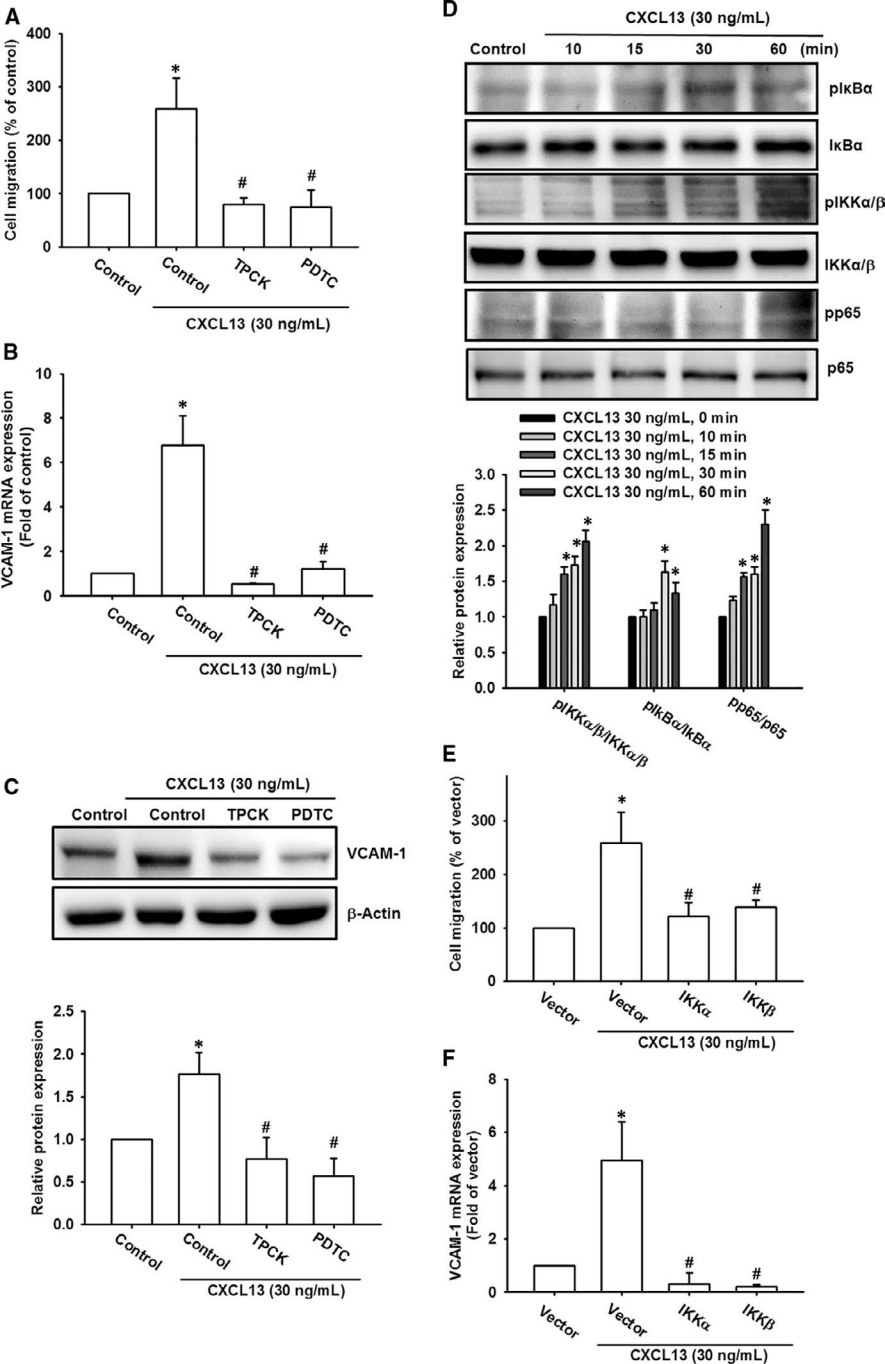
NF‐κB activation is responsible for CXCL13‐induced cell migration and VCAM‐1 expression in lung cancer cells. A‐C, The CL1‐0 lung cancer cells were pre‐treatment with pathway inhibitors of NF‐κB (PDTC, 5 μmol/L; TPCK, 5 μmol/L) for 1 h and then performed with cell migration assay A,, qPCR B, and Western blot C, in the presence of CXCL13 (30 ng/mL) for 24 h to evaluate cell migratory ability and VCAM‐1 expression, respectively. D, The CL1‐0 lung cancer cells were incubated with CXCL13 (30 ng/mL) for different time course (0, 10, 15, 30 and 60 min), the cell lysates were collected, and activation of NF‐κB signalling cascade was evaluated by monitoring phosphorylation of IKKα/β, IκBα and p65 proteins. The total proteins were used as internal control. E and F, The CL1‐0 lung cancer cells were transfected with dominant mutants of IKKα and IKKβ for 24 h and then performed with cell migration assay E, and qPCR F, in the presence of CXCL13 (30 ng/mL) for 24 h to evaluate cell migratory ability and VCAM‐1 expression, respectively. Results are expressed as the mean ± SD of triplicate samples. *P < .05 compared to the control group. #*P* < .05 compared to the CXCL13‐treated group

**FIGURE 7:**
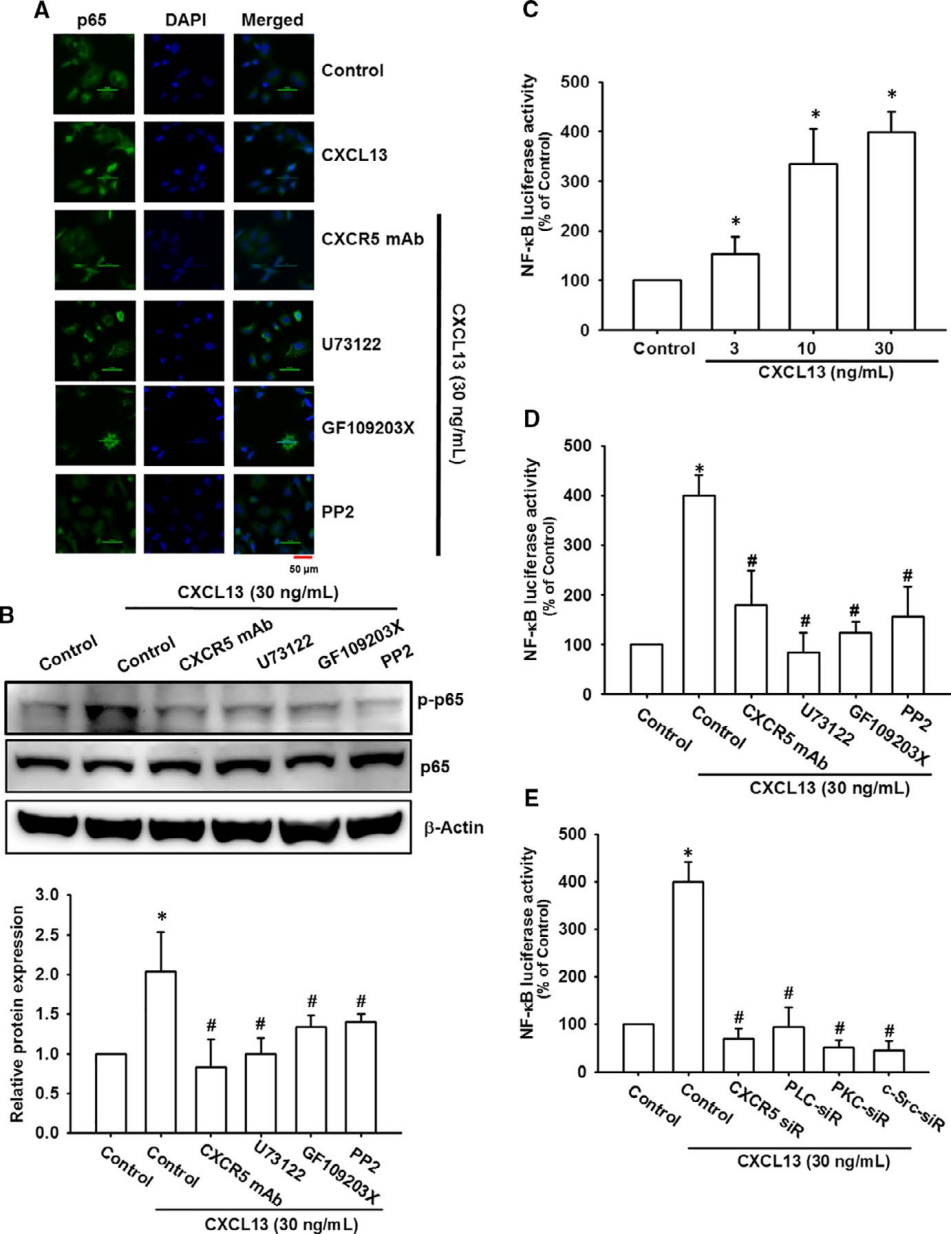
NF‐κB activation is downstream effector of CXCR5/PLCβ/PKCα/c‐Src signal cascade which contributes to VCAM‐1 expression and cell migration in lung cancer cells. (A and B) The CL1‐0 lung cancer cells were pre‐incubated with CXCR5 neutralized antibody (1 μg/mL), U73122 (0.5 μmol/L), GF109230X (3 μmol/L) or PP2 (5 μmol/L) for 1 h, follow by incubated with CXCL13 (30 ng/mL) for 24 h. Then, immunofluorescence staining was performed with anti‐p65 antibody. Nuclei were counterstained with DAPI. Representative microscopy images were shown A. The NF‐κB activation was monitored by phosphorylation of p65. The p65 and β‐Actin were used as internal control B. C, The CL1‐0 lung cancer cells were transfected with NF‐κB luciferase reporter vector for 24 h, follow by incubated with CXCL13 (30 ng/mL) for further 24 h. Then, luciferase activity assay was performed to examine NF‐κB activation. D, The CL1‐0 lung cancer cells were transfected with NF‐κB luciferase reporter vector for 24 h, follow by incubated with CXCR5 neutralized antibody (1 μg/mL), U73122 (0.5 μmol/L), GF109230X (3 μmol/L) or PP2 (5 μmol/L) for 1 h, then exposed to CXCL13 (30 ng/mL) for further 24 h. Finally, the luciferase activity was performed to examine NF‐κB activation. E, The CL1‐0 lung cancer cells were cotransfected with NF‐κB luciferase reporter vector in the presence of CXCR5, PLCβ, PKCα or c‐Src siRNAs for 24 h, follow by incubated with CXCL13 (30 ng/mL) for further 24 h. Then, luciferase activity assay was performed to examine NF‐κB activation. Results are expressed as the mean ± SD of triplicate samples. *P < .05 compared to the control group. #*P* < .05 compared to the CXCL13‐treated group

NF‐κB is a crucial transcription factor and its activation is correlated with cancer cell migration and invasion.^32^, ^33^ It is also a key downstream effector of chemokine receptor signalling.^31^ Therefore, we confirmed the activation of NF‐κB components after CXCL13 treatment in lung cancer cells. With pre‐treatment of NF‐κB inhibitors PDTC and TPCK, cell migration and VCAM‐1 were significantly abolished in CXCL13‐incubated lung cancer cells (Figure 6A‐C). Meanwhile, our results also showed that levels of phosphorylated p65, IKKα/β and IκBα were up‐regulated after 15‐60 minutes of CXCL13 treatment (Figure 6D). Inhibition of NF‐κB signal activation by dominant negative mutants of IKKα and IKKβ also decreased CXCL13‐promoted cell migration and VCAM‐1 expression (Figure 6E,F). We also checked whether CXCR5, PLCβ, PKCα and c‐Src were the upstream regulator of NF‐κB, with pre‐treatment of CXCR5 neutralized antibody, PLCβ, PKCα or c‐Src inhibitors, the CXCL13‐induced p65 nuclear translocation, representing for NF‐κB activation, was reversed by pre‐treatment with CXCR5 neutralized antibody, PLCβ, PKCα and c‐Src inhibitors (Figure 7A), as well as p65 phosphorylation (Figure 7B). Finally, to further examine the transcriptional activation of NF‐κB is responsible for CXCL13 effects, the luciferase reporter assay was performed. The NF‐κB activity was significantly increased after CXCL13 treatment (Figure 7C). However, in the presence with pathway inhibitors or siRNAs of CXCR5, PLCβ, PKCα and c‐Src, the NF‐κB activity was reversed after CXCL13 treatment (Figure 7D,E). In conclusion, these evidences suggest that the CXCR5, PLCβ, PKCα and c‐Src signalling pathways mediate NF‐κB activation in lung cancer cells treated with CXCL13.

## 4 DISCUSSION

CXC chemokine ligand‐13 and its corresponding receptor CXCR5 have been widely reported in various cancers.^34^ However, the prognostic value and pathological role of CXCL13/CXCR5 in lung cancer is still in its infancy. Here, we report the prognostic relevance of CXCL13/CXCR5 and found that they are commonly overexpressed among different subtypes of lung cancer. Previous report showed that high levels of CXCR5 and CXCL13 were up‐regulated in tissues and sera of lung cancer patients respectively.^35^ Here, we used online database to perform meta‐analysis, and results indicated CXCL13 and CXCR5 were highly expressed in different data sets. This evidence suggests that CXCL13/CXCR5 axis activation is common feature in lung cancer progression. Recently, the therapeutic applications by targeting CXCL13/ CXCR5 axis have been summarized.^34^ The strategies by using RNA interference and pharmacological intervention have been investigated in several studies and showed promising effects. Our present work reveals that CXCL13/CXCR5 axis could be a novel therapeutic target against lung cancer.

CXC chemokine ligand‐13, first identified as BLC (B‐lymphocyte chemoattractant) or BCA‐1 (B cell‐attracting chemokine 1), a homeostatic chemokine which attracting B lymphocytes while exhibiting a minor effect on T cells and macrophages.^36^ Previous studies have proved that CXCL13 promotes cancer cells migration via different biological molecules, for example matrix metalloproteinase‐9 (MMP9) ^37^ and MMP13.^38^ Here, we found that VCAM‐1, a cell adhesion molecule associated with metastasis, which was up‐regulated in response to CXCL13 in lung cancer cells. VCAM‐1 expression is reported to be closely implicated in the metastasis of many cancer cells. In breast cancer cells, VCAM‐1 expression was up‐regulated in the selected cancer cells which metastasized to lung.^39^ In glioblastoma, VCAM‐1 expression correlated with the clinicopathological grade of cancer.^8^ VCAM‐1 expression was also found to be up‐regulated in lung cancers.^9^ Here, we also investigated the correlation between CXCL13 and VCAM‐1 in tissues of lung cancer patients. The highly correlation between CXCL13 and VCAM‐1 supports our finding in in vitro experiments. As previous study, the indication of VCAM‐1 is a critical component requiring for lung cancer invasion and further metastasis.^9^ Our finding here supposes that CXCL13/CXCR5 axis is a major regulator of VCAM‐1 up‐regulation in lung cancer.

The signal activation downstream of CXCL13/CXCR5 axis has been discussed in several reports, and however, the whole scenario of CXCL13/CXCR5 is still poorly understood. In this study, we found PLCβ/PKCα/c‐Src signal cascade was activated after CXCL13 treatment in lung cancer cells. Furthermore, this activation was required for VCAM‐1 expression and then cell migration. As the G protein‐coupled receptors (GPCRs), which forming chemokine ligand/receptor pair axes, commonly activates PLC/diacylglycerol (DAG)/PKC signal transduction.^40^ PLC and PKC are important components of the phosphoinositide (PI) signalling system. The ability of PLC to produce DAG and IP3, and its proven capacity to activate classical PKCs in some cell types.^41^, ^42^ The PLC or PKC activation in response to CXCL13/CXCR5 axis has not been reported yet. This study first reports that CXCL13 stimulates PLCβ and PKCα which participating cell migration effect. Mountain of evidence has indicated Src as a critical molecule in cancer progression including cell survival, angiogenesis, migration, invasion and metastasis.^43^ Therefore, inhibition of Src activity provides potential strategy against tumour progression. Here, we found that Src was activated by CXCL13/CXCR5 axis and subsequently regulated cell migration in lung cancer cells. In accordance with Haibi et al^44^ reported in prostate cancer, CXCL13/CXCR5‐promoted cell migration and invasion was regulated by Src activation. In regard to the key role of Src in tumour progression, combination therapy of CXCL13/CXCR5 axis and Src inhibitors may provide clinical benefits in lung cancer treatment.

The transcription factor NF‐κB has been widely discussed in cancer progression and targeting NF‐κB as a therapeutic strategy has been explored extensively in the past decades. NF‐κB has been implicated in various cellular functions such as cell proliferation, apoptosis, angiogenesis, cell migration and invasion.^45^ Previous review has summarized that NF‐κB is a downstream signalling effector of G protein‐coupled receptor (GPCR), which is regulated by affecting upstream signal components or other transcription factors that coordinate with NF‐κB.^46^ However, the activation of NF‐κB by CXCR5, a member of GPCR super family, has never been reported yet. Our results indicated that NF‐κB signal pathway was activated by CXCL13/CXCR5 axis. Interestingly, previous reports have provided evidence that NF‐κB transcriptional activation was required for CXCL13 release or CXCR5 expression,^47^, ^48^ revealing the pivotal role of NF‐κB in CXCL13/CXCR5 axis activation. Combined with our finding here, the autocrine loop mediated by CXCL13/CXCR5 and NF‐κB may contribute to enhancement of cell migration effect.

In conclusion, our present study suggests that CXCL13/CXCR5 axis is up‐regulated in lung cancer cells, which promotes VCAM‐1 expression and further cell migration. We also decipher the molecular mechanism involved in regulation of this effect, which is achieved by PLCβ/PKCα/c‐Src signal cascade and NF‐κB transcription factor. Based on our finding here, the clinical relevance of CXCL13/CXCR5 in lung cancer progression and development of novel therapeutic targets should be addressed in the future.

## CONFLICT OF INTEREST

The authors declare no competing interests.

## AUTHOR CONTRIBUTIONS

**Chia‐Chia Chao:** Investigation (lead); Project administration (lead); Writing‐original draft (lead). **Wei‐Fang Lee:** Investigation (supporting); Project administration (supporting). **Shih‐Wei Wang:** Methodology (lead). **Po‐Chun Chen:** Methodology (lead). **Ayaho Yamamoto:** Methodology (lead); Writing‐original draft (lead). **Tsung‐Ming Chang:** Investigation (lead). **Shun‐Long Weng:** Funding acquisition (lead); Investigation (lead); Methodology (lead); Writing‐original draft (lead); Writing‐review & editing (lead). **Ju‐Fang Liu:** Project administration (lead); Supervision (lead); Validation (lead); Writing‐original draft (lead); Writing‐review & editing (lead).

## Data Availability

The data sets used and analysed during the current study are available from the corresponding author on reasonable request.
